# Biodegradable Polyurethane Derived from Hydroxylated Polylactide with Superior Mechanical Properties

**DOI:** 10.3390/polym16131809

**Published:** 2024-06-26

**Authors:** Xueqin Li, Yanyan Lin, Cengceng Zhao, Na Meng, Ying Bai, Xianfeng Wang, Jianyong Yu, Bin Ding

**Affiliations:** 1 Shanghai Frontier Science Research Center of Advanced Textiles, College of Textiles, Donghua University, Shanghai 201620, China; 1219718@mail.dhu.edu.cn (X.L.); yylin@mail.dhu.edu.cn (Y.L.); 1229720@mail.dhu.edu.cn (C.Z.); 15257305824@163.com (N.M.); yujy@dhu.edu.cn (J.Y.); 2 Innovation Center for Textile Science and Technology, Donghua University, Shanghai 201620, China; 3 Textile Industry Science and Technology Development Center, Beijing 100020, China; 15202136680@163.com

**Keywords:** polylactide-based polyurethane, biodegradation, soft segment, toughness

## Abstract

Developing biodegradable polyurethane (PU) materials as an alternative to non-degradable petroleum-based PU is a crucial and challenging task. This study utilized lactide as the starting material to synthesize polylactide polyols (PLA-OH). PLA-based polyurethanes (PLA-PUs) were successfully synthesized by introducing PLA-OH into the PU molecular chain. A higher content of PLA-OH in the soft segments resulted in a substantial improvement in the mechanical attributes of the PLA-PUs. This study found that the addition of PLA-OH content significantly improved the tensile stress of the PU from 5.35 MPa to 37.15 MPa and increased the maximum elongation to 820.8%. Additionally, the modulus and toughness of the resulting PLA-PU were also significantly improved with increasing PLA-OH content. Specifically, the PLA-PU with 40% PLA-OH exhibited a high modulus of 33.45 MPa and a toughness of 147.18 MJ m^−3^. PLA-PU films can be degraded to carbon dioxide and water after 6 months in the soil. This highlights the potential of synthesizing PLA-PU using biomass-renewable polylactide, which is important in green and sustainable chemistry.

## 1. Introduction

Polyurethane (PU) is a unique type of polymer that distinguishes itself from other plastic in several ways [1,2,3,4]. It can be widely used in automotives, aviation, electronics, building materials, agriculture and other areas due to its advantages of light weight, easy processing and corrosion resistance [4,5,6,7,8,9,10]. However, the recycling of waste plastics is challenging and often not cost-effective [11]. Due to the continuous increase in fossil energy consumption and the growing awareness of environmental protection, renewable biodegradable materials are becoming increasingly important as alternatives to petroleum-based plastics for the sustainable development of industry and human society [12,13]. High-performance biobased polyurethanes (Bio-PUs) have a wide range of applications, including electronic devices [14,15], tissue engineering [16,17,18], agricultural film [19], express packaging [20], food packaging and other fields [21,22,23].

Polylactide (PLA) is a popular biodegradable and biocompatible polymer [24,25,26,27]. It is a biobased aliphatic polyester that can serve as a soft segment of PU; PLA is produced through two methods: the ring-opening polymerization of lactide and dehydration condensation reaction of lactic acid molecules [28,29,30]. It has been discovered that the crystallinity of PLA has a great influence on its mechanical properties, thermal characteristics and degradation mechanism [31]. Adding PLA can significantly enhance the mechanical strength and degradability of aliphatic PU, as previously mentioned. However, PLA may not be suitable for single-component soft segments in PU. Gu et al. [32] synthesized PLA-based polyurethanes (PLA-PUs) with 1,6 hexamethylene diisocyanate (HDI), and the prepared samples had a high modulus of elasticity (about 850 MPa); however, the elongation at break was low at only 4%. Thus, the mechanical strength and elasticity of PU cannot be balanced by using only PLA diols as soft segments. The combined employment of a rigid PLA segment and a flexible part seems to be a viable solution for the manufacture of PU. Shi et al. [33] prepared a series of PU elastomers using PLA and polytetramethylene glycol (PTMG) as the two-component soft segment, and HMDI was the hard segment. Through this molecular coordination, PTMG provides elasticity, and the soft segment of PLA imparts better strength to the PU. However, poly (ethylene glycol) adipate (PEA) exhibits more excellent flexibility, heat resistance, mechanical strength and other properties than PTMG, which can be used with PLA as a soft segment. In this way, we can control the thermal and mechanical properties of PU by adjusting the quality of two different soft segments [34,35].

This work presents a novel PLA-PU with high strength and excellent toughness. PLA and PEA polyols were used as the soft segments, while 4,4-methylene diphenyl diisocyanate (MDI) and ethylene glycol (EG) acted as the hard segments. The investigation focused on the influence of varying the polylactide polyol (PLA-OH) content in soft segments on the hydrogen bonding analysis, mechanical properties and thermal properties of the PLA-PUs. The tensile test showed a stress of 37.15 MPa and an elongation of 820.8%. Additionally, they exhibited a high modulus of 33.45 MPa and a toughness of 147.18 MJ m^−3^. PLA-PU materials were biodegradable after disposal and degraded more than 80% within 6 months. Its decomposition products carbon dioxide (CO_2_) and water (H_2_O) can be recycled by plants [36]. The synthesized PLA-PUs exhibit good biodegradability and mechanical properties when compared to commercial PUs. This makes them suitable for use in biomaterials, green packaging, consumer products, building materials, etc.

## 2. Materials and Methods

### 2.1. Materials

L-lactide was purchased from Fengyuan Biotechnology Co., Ltd. (Bengbu, China). EG, MDI and PEA with 2000 molecular weight were purchased at TCI Development Co., Ltd. (Shanghai, China). Diethylene Glycol (DEG), Stannous octanoate (Sn(Oct)_2_), methanol (CH_3_OH), trichloromethane (CHCl_3_), tetrahydrofuran (THF), N, N-dimethyl formamide (DMF) and dibutyltin dilaurate (DBTDL) were obtained from Aladdin Chemistry Co., Ltd. (Shanghai, China).

### 2.2. Synthesis of PLA-OH and PLA-PUs

**Scheme 1:**
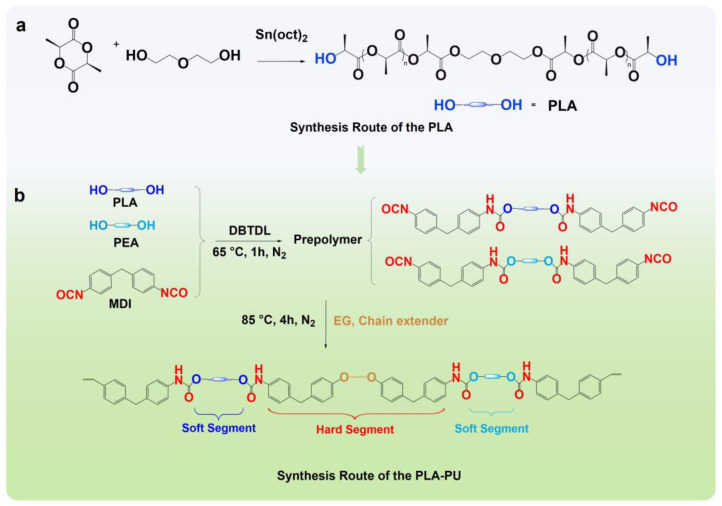
(**a**) Synthesis route of PLA-OH. (**b**) Synthesis route of PLA-PUs from PLA-OH, PEA polyols, MDI and EG.

Hydroxylated PLA-OH was synthesized using L-lactide as the starting material by the ring-opening method (Scheme 1a), and the preparation process was simple, achieving a conversion rate of over 95%. Firstly, L-lactide and DEG in a molar ratio of 13:1 were added to a 500 mL four-necked flask, followed by gradual heating from ambient temperature to 120 °C. The mixture was completely melted under mechanical stirring. Following that, Sn(Oct)_2_ was introduced as a catalyst, and the reaction temperature was maintained at 160 °C for 4 h. Subsequently, the resultant product was dissolved in CHCl_3_ and purified by precipitation with an excess of cold CH_3_OH. After drying the refined product at 70 °C for 48 h, high-purity PLA-OH (relative molecular weight 2000 g/mol) was obtained.

The preparation of PLA-PUs is described in Scheme 1b. Firstly, the prepolymer was prepared by combining the PLA-OH, PEA, MDI, DBTDL (0.03 wt%) and an appropriate amount of DMF in a four-neck flask with a capacity of 500 mL, and the reaction took place at 65 °C for 1 h. The molar ratio of the OH group (from PLA-OH, PEA polyol and EG) to the NCO group (from MDI) for all samples was fixed as 1:1, and the detailed formulation is shown in Table S1. To obtain high-molecular weight PLA-PUs, a chain extension process was employed with EG as the chain extender; the soft segment components of PEA and PLA were randomly and uniformly arranged. After the reaction, the polymers were placed inside an oven at 100 °C for 10 h (curing took place for 8 h at atmospheric pressure, followed by an additional 2 h at reduced pressure to remove DMF). To fabricate films from the PLA-PUs, they were pressed using a four-sided film applicator. The thickness of the films was carefully controlled at 0.5 ± 0.1 mm. PLA-PUs with varying contents of PLA-OH were synthesized and labeled as 0% PLA-PU, 10% PLA-PU, 20% PLA-PU, 30% PLA-PU and 40% PLA-PU, respectively. The x% denotes the mass of PLA-OH in the soft segment of the polymer. Table S2 summarizes the molecular weight of PLA-PU, and the hard segment in all samples was controlled to be between 25 and 30 wt% to ensure consistency.

### 2.3. Characterizations

The number average molecular weight (Mn), weight average molecular weight (Mw) and polydispersity index (PDI) of the PLA-PUs were investigated by Gel permeation chromatography (GPC, Agilent PL-GPC220) utilizing a stainless steel column suitable for the separation of lipophilic organic compounds within the molecular weight range of 10^2^ to 10^7^ Daltons. Monodisperse polystyrene was used as the calibration standard for molecular weight determination. Specifically, 50 mg of PLA-PU was dissolved in 5 mL of THF as the mobile phase at a flow rate of 1 mL min^−1^.

Nuclear magnetic resonance (NMR) spectra were recorded on a Bruker ARX400 (Wissembourg, France) was used to determine the chemical structures of the PLA-OH and PLA-PUs with deuterochloroform as the solvent.

The Fourier transform infrared (FTIR) spectra were recorded using attenuated total reflection (ATR) in transmission mode with a PARAGON 1000 PerkinElmer FTIR Spectrophotometer (Waltham, MA, USA).

The thermal decomposition characteristics of PLA-PUs were evaluated by a thermogravimetric analyzer (TGA) was performed on TGA, 209 F1 (NETZSCH, Waldkraiburg, Germany).

The thermal properties and crystallization characteristics of PLA-PUs were examined using a differential scanning calorimeter (DSC) from PerkinElmer (Waltham, MA, USA). Initially, the sample underwent a heating scan from 30 °C to 200 °C to eliminate any previous thermal history. Subsequently, the samples were cooled to −70 °C and then heated again from −70 °C to 200 °C. Finally, the samples were cooled again to 40 °C. All samples were scanned under N_2_ atmosphere at a rate of 10 °C min^−1^. The DSC analysis provided insights into the thermal transitions, such as melting and crystallization during the heating and cooling cycles. Both the first cool-down and second warm-up data were recorded for all samples.

Tensile tests were carried out using a universal tensile strength instrument (YG026G-III, Wenzhou Fangyuan Instrument Co., Ltd., Wenzhou, China). The films were cut into a size of 15 × 10 × 0.5 mm and stretched at a rate of 30 mm min^−1^. Each sample was measured at least five times. Only the breaking strength of the PLA-PU films could be measured during the tests. The stress of PLA-PUs was calculated using the formula σ = F/(l × d), where σ, F, l and dare the stress, breaking strength, width and thickness of samples, respectively. The stress experienced by the PLA-PU films during the tensile tests was determined through this calculation.

The ultraviolet–visible (UV-Vis) absorption spectra of the samples were measured on a UV-Vis spectrometer (U-T9S, Shanghai Yipu Instrument Co., Ltd., Shanghai, China) under a wavelength of 300–800 nm. This analysis provided information about the UV-Vis absorption properties of the PLA-PU films in the specified wavelength range.

### 2.4. Degradation Performance Test

To examine the outdoor biodegradability of PLA-PU films, 5 × 5 cm^2^ PLA-PU film samples were buried 10 cm below the soil surface (~22 °C, City of Shanghai, 30°40′ N, 120°52′ E). The samples were excavated monthly, cleaned to remove any soil particles and dried. The morphologies and structural changes in the PLA-PUs were observed using a VEGA III Scanning Electron Microscope (SEM, TESCAN Ltd., Brno-Kohoutovice, Czech Republic).

## 3. Results and Discussion

### 3.1. Chemical Structure of PLA-OH and PLA-PUs

**Figure 1:**
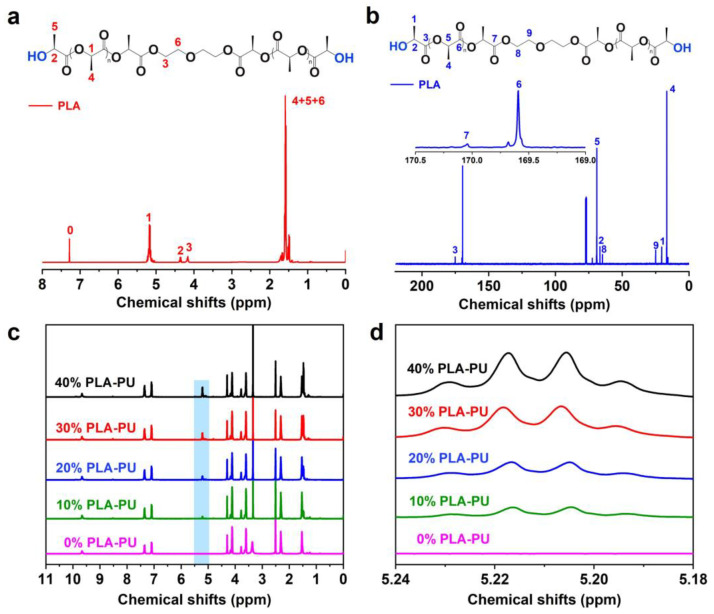
(**a**) The chemical structure of PLA-OH and its corresponding ^1^H NMR and (**b**) ^13^C NMR chemical shifts (the inset shows an enlargement of the chemical shifts from 169 to 170.5 ppm), (**c**) The ^1^H NMR of PLA-PUs in the organic phase. (**d**) The ^1^H NMR spectra (enlarged chemical shift from 5.0 to 5.5 ppm) of PLA-PUs in the organic phase.

The ^1^H NMR spectrum of the PLA-OH is shown in Figure 1a (the numbers represent chemical shifts and correspond to the numbers next to the structural formula of the molecule) and Figure S1. The characteristic chemical shift 0 was the solvent peak of deuterochloroform (at 7.26 ppm). The characteristic chemical shifts 1 (at 5.16 ppm) and 4 (at 1.59 ppm) were related to the CH and CH_3_ of the lactide repeat units, respectively, and the chemical shifts 2 (at 4.36 ppm) and 3 (at 4.16 ppm) could be assigned to the CH of the end lactide repeats units of the chain and the outer CH_2_ groups of DEG in the chain, respectively. Based on the above assignments, the molecular weight (Mn) of the synthesized PLA-OH diol could be calculated as follows:

$$\mathrm{Mn}=\left(\frac{I_{1}}{I_{2}}+1\right)\times 72\times 2+104$$

where *I*_1_ and *I*_2_ are the areas of peaks 1 and 2, respectively, 72 is the molar mass of one lactide repeat unit and 104 is the total molar mass of the rest of the molecule.

In order to verify that the synthetic PLA-OH diol is hydroxyl-terminated, the following was conducted. A ^13^C NMR spectrum of PLA-OH is shown in Figure 1b. The chemical shifts 8 (64.87 ppm) and 9 (25.09 ppm) were related to the −CH_2_CH_3_ of the DEG main chain; the chemical shifts 7 (170.09 ppm) can be assigned to the C of −O−CO− close to DEG; the chemical shifts 4 (16.7 ppm), 5 (69.05 ppm) and 6 (169.69 ppm) can be assigned to the C on the repeating unit in the PLA-OH main chain segment. The chemical shifts 1 (20.58 ppm), 2 (66.76 ppm) and 3 (175.15 ppm) can be assigned to the carbon atoms in the terminal lactic acid unit, respectively. The NMR carbon spectra demonstrated that the hydroxyl-terminated PLA-OH was successfully prepared. The ^1^H NMR spectra of PLA-PUs are presented in Figure 1c,d. The chemical shifts at 9.7 ppm may belong to the carbamate group, while those of the −CH_2_− groups are observed at 1.67, 2.35, 3.89, 4.14 and 4.36 ppm and the chemical structures of PEA diol at 3.43 ppm (Figure 1c). The chemical shift of PLA-OH (blue bar area) was found at 5.17 ppm (Figure 1d). It demonstrates that the PLA and PEA segments were successfully incorporated in the main chain.

The Mw of the PLA-PU samples were also investigated by GPC. The results, including the Mn, Mw and PDI, are listed in Table S1. It was observed that as the content of PLA-OH in the soft segment increased, the Mn of the PLA-PUs gradually decreased, and the Mw increased. This may be due to the fact that PLA-OH chains are difficult to align regularly, and when PLA-OH is added, it reduces the regularity of the material, which hinders an increase in the Mn of the PLA-PUs. It is well known that higher Mw values for PLA-PUs result in improved mechanical properties [37,38,39,40]. This is probably due to the fact that the molecular chain is easily arranged regularly when the content of PLA-OH is low, and as the content of PLA-OH increases, the regularity of PU decreases, but the Mw increases, which may be due to the fact that PLA-OH increases the reticulation of the physical cross-linking of PU and the entanglement of the PU chain, which leads to an increase in the reticulation rigidity of the PU chain, and the PLA-OH rigidity structure occupies a dominant position in the molecular chain of PU, which then affects the mechanical properties of the molecular chain of PU. The polydispersity index (PDI) values indicating the molecular weight distribution were found to be relatively low, ranging from 1.81 to 1.92. The low PDI values imply a narrow distribution of the Mw in the PLA-PUs. In conclusion, the analysis presented above demonstrates the successful synthesis of PLA-OH and PLA-PU and provides insights into the relationship between the content of PLA-OH and PLA-PUs.

### 3.2. FTIR Analysis of PLA-OH and PLA-PUs

**Figure 2:**
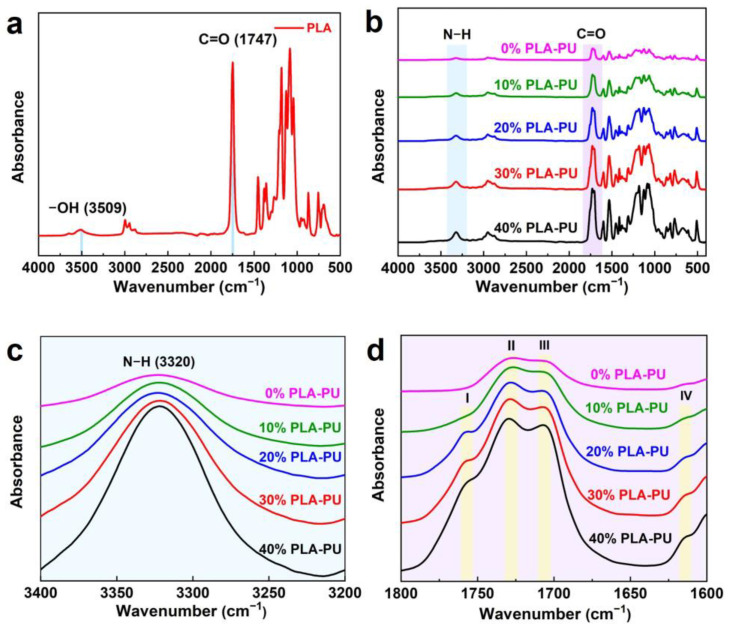
(**a**) FTIR spectra of PLA-OH and (**b**) PLA-PUs, (**c**) N-H group at 3320 cm^−1^ enlargement of PLA-PUs. (**d**) C=O group at 1600~1800 cm^−1^ enlargement of PLA-PUs.

Figure 2a presents the characteristic peak of PLA-OH, including C=O (1747 cm^−1^) and −OH (3509 cm^−1^). The characteristic peak at 3509 cm^−1^ disappeared (Figure 2b), which means that PLA-OH was completely depleted in the reaction system [41]. The −NCO peak of MDI at 2270 cm^−1^ disappeared. It was confirmed that all the −NCO were totally reacted and formed carbamate bonds throughout the polymerization process [42]; the broad absorption band observed near 3300 cm^−1^ and 1700 cm^−1^ corresponds to the N−H (blue bar area) and C=O (purple bar area) vibrations of the urethane linkage (Figure 2b). It is well established that the N−H group serves as a proton donor, while the C=O group functions as a proton acceptor [43,44,45]. The presence of hydrogen bonds can be inferred from the N−H group at 3320 cm^−1^ (Figure 2c). The PLA-PU sample exhibited four absorption peaks (yellow bar area) for the C=O groups (Figure 2d), which are peaks I (1757 cm^−1^), II (1730 cm^−1^), III (1706 cm^−1^) and IV (1679 cm^−1^). To investigate the hydrogen bonds in the samples, peaks were fitted to the C=O region of the samples by infrared peak-splitting software (PeakFit V4.12). It is evident that in the 0% PLA-PU sample, only peaks II and III were observed (Figure S2a), which should be assigned to the free carbonyl group and the hydrogen-bonded carbonyl group in the hard domains, respectively. However, with the addition of PLA diol, peaks I and IV appear, and the intensities of the absorption peaks increase with the increasing content of PLA (Figure 2d). Peak I at 1757 cm^−1^ can be assigned to those free of hydrogen bonding, while the hydrogen-bonded ordered carbonyl groups are between 1730 and 1679 cm^−1^. The bonded C=O content in 40% PLA-PU was determined to be as high as 84.1% (Figure S2b), which was much higher than those of 0% PLA-PU (55.2%); peak IV at 1679 cm^−1^ is associated with hydrogen-bonded ones. The free hard segment might disperse in the soft matrix and form hydrogen bonding with the C=O group of the PLA component, which is manifested by peak IV. However, the compatibility between the hard segment and PLA domain was very limited, and a lot of C=O groups remained free.

### 3.3. Thermal, Mechanical and Optical Properties of PLA-PUs

**Figure 3:**
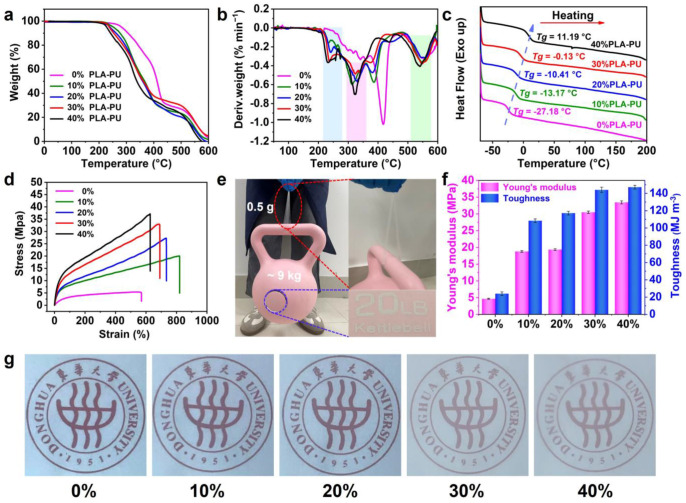
(**a**) Weight loss curves and (**b**) their derivatives of PLA-PUs. (**c**) Tg values of PLA-PUs determined by DSC. (**d**) Tensile stress–strain curves. (**e**) Display of mechanical properties of 40% PLA-PU film. (**f**) Young’s modulus and toughness of PLA-PUs. (**g**) Digital pictures (thickness of film is ~0.5 mm, closely attached to logo of Donghua University, Shanghai, China). Use of logo is permitted with permission by Donghua University.

The samples were investigated using TG and DSC. All PLA-PUs were thermally stable without noticeable weight loss until 230 °C (Figure 3a). PLA-OH begins thermal decomposition at 180 °C (Figures S3 and S4). It was observed that the addition of PLA-OH led to a decrease in the stability of the thermal properties of PU. It was also found that the more the content of PLA-OH, the worse the thermal stability of PU, which may be due to the fact that when PU undergoes thermal decomposition, the PLA chain in the main chain of PU is the first to start thermal decomposition, and then the other chain segments of PU start to decompose gradually, so it leads to the phenomenon that the higher the content of PLA-OH, the worse the thermal stability of PU. According to the thermal decomposition diagram, the thermal degradation of PLA-PU had three distinct stages (Figure 3b). In the first stage (blue bar area), the weight loss of the sample remained below 25% as the temperature increased from 230 to 250 °C. The second stage (pink bar area) witnessed a significant decline in the sample’s thermal stability, resulting in a weight loss exceeding 80% as the temperature from 300 to 350 °C. However, PU without the PLA-OH soft segment did not exhibit this rapid degradation. This is attributed to the thermal degradation of the main chemical structures of PLA in the soft segment. The final stage (green bar area) occurred between 520 °C and 580 °C with less weight loss, where all polyurethanes show similar thermal degradation behavior, as shown in the green area of Figure 3b, which may be due to the thermal degradation of the chemical structure of the hard segments.

Figure 3c and Figure S5 show the DSC second heating and first cooling curves, respectively. The thermodynamic incompatibility among the soft and hard segments of PU was widely recognized as a key factor affecting the extent of microphase separation [46]. The 0% PLA-PU sample exhibited a lower glass transition temperature (Tg), and PLA-OH increased the values for 10% PLA-PU, 20% PLA-PU, 30% PLA-PU and 40% PLA-PU to −13.17, −10.41, −0.13 and 11.19 °C, respectively (Figure 3c). Due to the low Mw (Table S2), the molecular chain lengths of the soft and hard segments are not sufficient to form highly ordered soft and hard areas. Thus, PLA-PUs behaved as an amorphous state, without any melting heat absorption that was observed in the first cooling curves (Figure S5). The variation in Tg ranged for PLA-PUs from −27.18 to 11.19 °C, which is between the Tg of PEA (−49.09 °C) and PLA (23.71 °C), which further indicated the hybrid of hard-soft segment phase in the PU (Figure S6). No Tg associated with the hard segment was detected in the DSC curve (Figure 3c). It may be that MDI contains benzene rings with short chain segments, which can significantly inhibit chain stacking in hard segments. In addition, the Tg of PU monotonically increased with the increasing content of PLA and gradually approached the corresponding temperatures of neat PLA diol. Such a phenomenon implied good microphase separation between the PLA segment and other components. The Tg of the hard domain was not found in the DSC experiment, which might imply the weak microphase separation of hard segments. This indicates that the increase in the PLA-OH chain content promotes the formation of larger phase regions in PLA chain segments, improving the extent of the microphase segregation of PU.

We also examined the mechanical properties of PU samples with varying amounts of PLA-OH. It can be clearly seen that the introduction of PLA-OH segments into the polyurethane main chain significantly enhanced the mechanical rigidity of the PU material (Figure 3d). Approximately a weight of 9 kg can be lifted using only 0.5 g of the 40% PLA-PU film (Figure 3e). With an increase in the proportion of PLA-OH, the PU material resulted in a continuous enhancement in Young’s modulus and toughness (Figure 3f). Notably, the 40% PLA-PU demonstrated superior performance, exhibiting a remarkable Young’s modulus of 33.45 MPa and an impressive toughness value of 147.18 MJ m^−3^, which are far superior to 0% PLA-PU (Figure 3f). However, the material’s elongation at break consistently declined (Figure S7). The continuous increase in the content of PLA-OH may promote the crystallization of PLA chain segments and the formation of more organized phase domains [47]. During the early stage of stretching, the higher the perfection in the structure of these phase domains, the greater the tensile stress they can withstand, resulting in a continuous increase in the material’s Young’s modulus [48]. During the later stage of stretching, the original phase structure of PU was destroyed, and the PLA chain segments were oriented along the stretching direction [33,49]. Due to the high Tg of PLA-OH, these oriented structures underwent significant changes in the course of the stretching process. Consequently, as the content of the PLA-OH increased, the resistance to tension of PU also increased. The maximum resistance to tension of 40% PLA-PU was 37.15 MPa (Figure S7). However, the sliding of the PLA-PU segments during the tensile process was hindered due to the poor compatibility between PLA segments and other constituents in PU. This resulted in a gradual reduction in the fracture elongation of PLA-PU as the PLA-OH content increased. The stress–strain curves of all PLA-PUs can be divided into three stages: the (1) Hookean elastic region, (2) strain region and (3) breaking region [9,50,51,52,53]. To provide insight into the mechanical performance of PLA-PUs at three different stages, we recorded the whole stretching process of 0−40% PLA-PUs with different times (Figure S8). As the PLA-OH chain content increased, the stretching time required to break the film gradually reduced at a higher tensile strength.

The optical property of polyurethane materials is an essential index for evaluating their sun resistance or aging resistance [54,55,56]. In UV-Vis testing, the transmittance of PLA PUs was measured and is presented in Figure S7. PLA-PUs with 0–20% PLA exhibited good transmittance in the visible light spectrum of 400–800 nm, with a transmittance of approximately 80%. However, when the PLA-OH content increased to 30–40%, it exhibited better shielding capabilities for UVA (320−400 nm) and UVB (300−320 nm). The 30–40% PLA-PU samples blocked nearly 100% of UVB and most of UVA [57]. The transparency of the film was negatively affected by the higher PLA-OH content (Figure 3g). It is evident that the content of PLA-OH has a significant impact on the transparency of the film. However, the results demonstrated that when the PLA-OH content was kept at 30% or above, the PLA-PU films had better shielding capabilities, indicating superior anti-aging performance.

### 3.4. Biodegradation Performance of PLA-PUs

**Figure 4:**
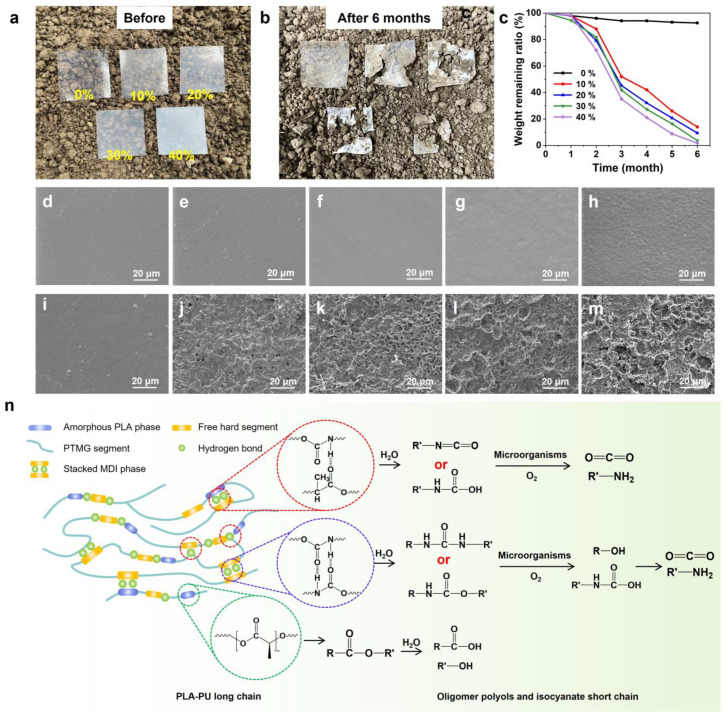
Digital photographs of PLA-PUs (**a**) before and (**b**) after 6 months of degradation in natural environment. (**c**) Weight variation in PLA-PUs during degradation. SEM images of (**d**) 0% PLA-PU, (**e**) 10% PLA-PU, (**f**) 20% PLA-PU, (**g**) 30% PLA-PU and (**h**) 40% PLA-PU before degradation. (**i**) Samples 0% PLA-PU, (**j**) 10% PLA-PU, (**k**) 20% PLA-PU, (**l**) 30% PLA-PU and (**m**) 40% PLA-PU after 6 months of degradation. (**n**) Degradation mechanism diagram of PLA-PUs.

The biodegradation performance of PLA-PUs is closely related to their composition. To compare, all PLA-PUs were buried in the same soil environment for biodegradation experiments. Characterization was conducted on the surface morphology, molecular weight and mass loss upon the degradation of dried PLA-PU samples. Observationally, all PLA-PU films exhibited smooth and transparent surfaces before degradation (Figure 4a). PU with PLA-OH underwent degradation in soil after a burial period of 6 months, and a higher content of PLA-OH resulted in a corresponding acceleration of degradation (Figure 4b). However, the control group, which consisted of the 0% PLA-PU material, showed minimal degradation with negligible alterations to its quality (Figure 4c). The hydrolysis of PLA-PU occurs through the random breakage of ester bonds, and PLA chain segments with a large number of ester groups preferentially undergo chain breakage, which is triggered by the diffusion of water in the amorphous region of the polymer. The degradation of PLA-PUs was slower during the initial two months. Primarily due to surface degradation processes, surface degradation occurs when the rate of hydrolysis exceeds the rate of water diffusion into the bulk or when the enzyme catalyst fails to penetrate the bulk polymer. It starts from the surface of the polymer; the volume of the polymer decreases. The degradation time reached the third or fourth month; further overall degradation occurs when the diffusion of water exceeds the rate of the hydrolysis reaction, and the degradation of PLA-PUs accelerated significantly.

The degradation process was mainly attributed to the breakdown of the polyester groups on the main chain of the material. The degradation of polyester, specifically PLA-OH, is an autocatalytic process. The hydrogen ions generated by hydrolysis can catalyze the deterioration of the remaining material. However, the degradation causes the polyester content on the surface of the material to decrease rapidly, resulting in a slow degradation in the next 5−6 months (Figure 4c). These phenomena suggest that microorganisms in the soil (e.g., bacteria and fungi) secrete enzymes to attack and digest PLA macromolecules and that this degradation occurs mainly on the ester bonds formed by PLA modification. The cleavage of ester bonds (−O−CO−, mainly in PLA) may lead to the disruption of the material’s three-dimensional structure and a reduction in its molecular weight. Therefore, the more PLA-OH content, the larger the area of degradation of the material. As the degradation occurs, the Mw of the material continues to decrease (Table S3), and the material basically loses its usability. The GPC test showed that the Mn of 40% PLA-PU decreased from 2.89 to 1.39 and 1.01 (×10^4^) after degradation for 1, 3 and 6 months. This further demonstrated that biodegradable PLA-PU materials maintain good mechanical properties during use and degrade rapidly at the end of their service life, reducing environmental impact.

To gain a deeper understanding of the degradation mechanism of the sample, the surface morphology of the PLA-PU film was examined before and after degradation using SEM. All samples had a smooth surface before degradation (Figure 4d–h). After 6 months of natural soil degradation, more aberrant and irregular pores were observed, and with the increase in PLA-OH content, the irregular pores became deeper and larger (Figure 4i–m). As the deterioration time increased to approximately 6 months, it was observed that 40% PLA-PU degraded the fastest. This was due to the degradation process moving from the surface to the inside of the film, resulting in wider cracks and more cavities. These results clearly indicated that even with a small amount of PLA-OH, enzymatic hydrolysis or hydrolysis can occur over time under the action of microorganisms.

There are many types of chemical degradations of PU, including hydrolysis, thermal degradation, thermal oxidative degradation, ultraviolet degradation, microbial degradation, etc. Microbial degradation is particularly likely to occur in natural environments. It is important to note that the ester group, carbamate group and urea group are the most susceptible to microbial degradation in PU. During PU degradation, urea formate, biuret and urea groups derived from isocyanate may form. Urea formate is formed through the reaction between isocyanate and formic acid, resulting in the replacement of one of the urethane linkages with a urea group (R–NH–CO–NH–COO–R’). Biuret is formed when isocyanate reacts with itself, leading to the formation of a cyclic compound with two urea groups (R–NH–CO–NH–CO–NH–R’). Urea can also be formed via the hydrolysis of urethane linkages (R–NH–COO–R’). These additional degradation products contribute to the complexity of the degradation process of PLA-PUs (Figure 4n). The degradation of PLA-PU polymer chains primarily involves the cleavage of ester linkages (R–COO–R’), predominantly located in PLA), R–NH–COO–R’ and the urea group (R–NH–CO–NH–COO–R’) (Figure 4n). R–COO–R’ underwent degradation to form carboxylic acid (R–COOH) and alcohol (R’–OH). The initial degradation of R–NH–COO–R’ leads to the generation of isocyanate (R–NCO) or carboxylic acid (R–NH–COOH) and alcohol (R’–OH), which further undergo degradation to produce CO_2_ and amine (R–NH_2_). The initial degradation of R–NH–CO–NH–COO–R’ results in the formation of R–NH–CO–NH–R’ and R–NH–COO–R’, which further degrade to R–NH–COOH and R’–OH. Finally, R–NH–COOH ultimately degrades to CO_2_ and R–NH_2_ [58]. In conclusion, PLA-PUs demonstrate excellent degradation properties, and the degradation products are not polluting to the environment.

## 4. Conclusions

This study synthesized a series of polyurethanes based on PLA, containing varying amounts of PLA diols and PEA as the soft components. The resulting materials exhibited high strength and exceptional toughness. As the PLA diol content increased in the soft segment, the resulting Tg of PLA-PUs increased. Simultaneously, PLA-PUs exhibited significant improvements in stress (37.15 MPa, increased by 7 times), elongation (820.8%, increased by 44%), toughness (147.18 MJ m^−3^, increased by 6 times) and Young’s modulus (33.45 MPa, increased by 7 times). Furthermore, PLA-PU is environmentally friendly and can degrade into CO_2_ and H_2_O in soil. It is foreseen that biodegradable PLA-PUs could potentially replace existing petroleum-based materials. This study highlights the potential of PLA-derived polyols as sustainable raw materials, capable of replacing petroleum-based polyols in PU synthesis. These new materials may offer tunable degradation rates and excellent mechanical properties, contributing to green sustainability efforts.

## Data Availability

The original contributions presented in the study are included in the article/supplementary material, further inquiries can be directed to the corresponding authors.

## Supplementary Materials

The following supporting information can be downloaded at https://www.mdpi.com/article/10.3390/polym16131809/s1. ^1^H NMR spectra of PLA-OH (Figure S1). The FTIR spectra of the 0% and 40% PLA-PU in the C=O absorption bands (Figure S2). TG curves of PLA (Figure S3). DTG curves of PLA (Figure S4). DSC first cooling curves of PLA-PUs. (Figure S5). DSC second heating curves of PLA and PEA. (Figure S6). The tensile strength and breaking elongation of PLA-PUs (Figure S7). The whole stretching process of 0–40% PLA-PUs with different times (Figure S8). The transmittance of PLA PUs in the wavelength of 300–800 nm (Figure S9). The formulation of the samples (Table S1). The molecular weights of PLA-PUs determined by GPC (Table S2). The molecular weight of PLA-PUs after degradation within 6 months determined by GPC (Table S3).
