# Supplementary material for: Biodegradable Polyurethane Derived from Hydroxylated Polylactide with Superior Mechanical Properties
Source: Polymers (Basel). 2024 Jun 26;16(13):1809. doi: 10.3390/polym16131809 (PMC11243797; doi:10.3390/polym16131809)
Supplement: Supplementary file 1 [file polymers-16-01809-s001.zip › polymers-3038226-supplementary.pdf]

Supporting Information:

Biodegradable Polyurethane Derived from Hydroxylated Polylactide with Superior Mechanical Properties

Xueqin Li [1], Yanyan Lin [1,2], Cengceng Zhao [1], Na Meng [1], Ying Bai [3], Xianfeng Wang [1,2,*], Jianyong Yu [1,2] and Bin Ding [1,2,*]

[1] Shanghai Frontier Science Research Center of Advanced Textiles, College of Textiles, Donghua University, Shanghai 201620, China; 1219718@mail.dhu.edu.cn (X.L.); yylin@mail.dhu.edu.cn (Y.L.); 1229720@mail.dhu.edu.cn (C.Z.); 15257305824@163.com (N.M.); yujy@dhu.edu.cn (J.Y.)

[2] Innovation Center for Textile Science and Technology, Donghua University, Shanghai 201620, China

[3] Textile Industry Science and Technology Development Center, Beijing 100020, China; 15202136680@163.com

* Correspondence: wxf@dhu.edu.cn (X.W.); binding@dhu.edu.cn (B.D.)

The supporting information contains:

Figures S1−S9

Tables S1−S3

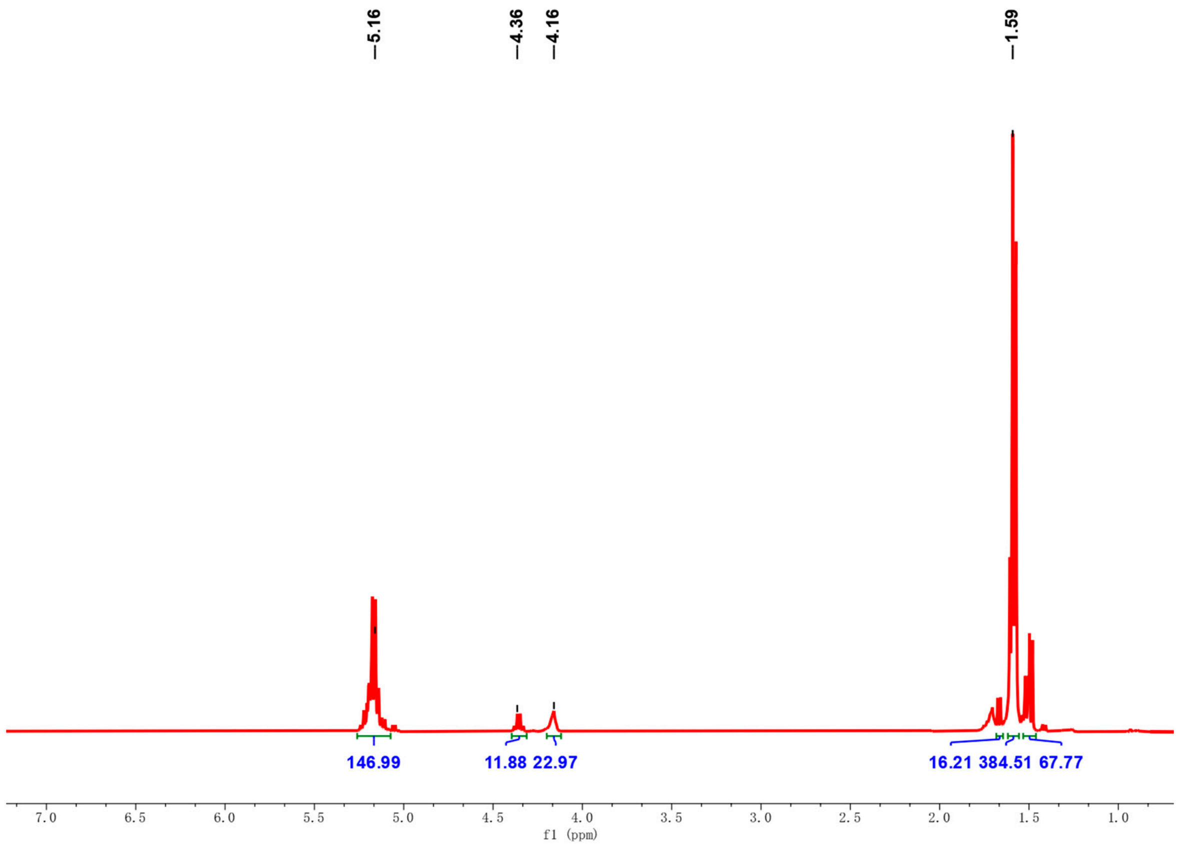

Figure S1. ^{1}H NMR spectra of PLA-OH.

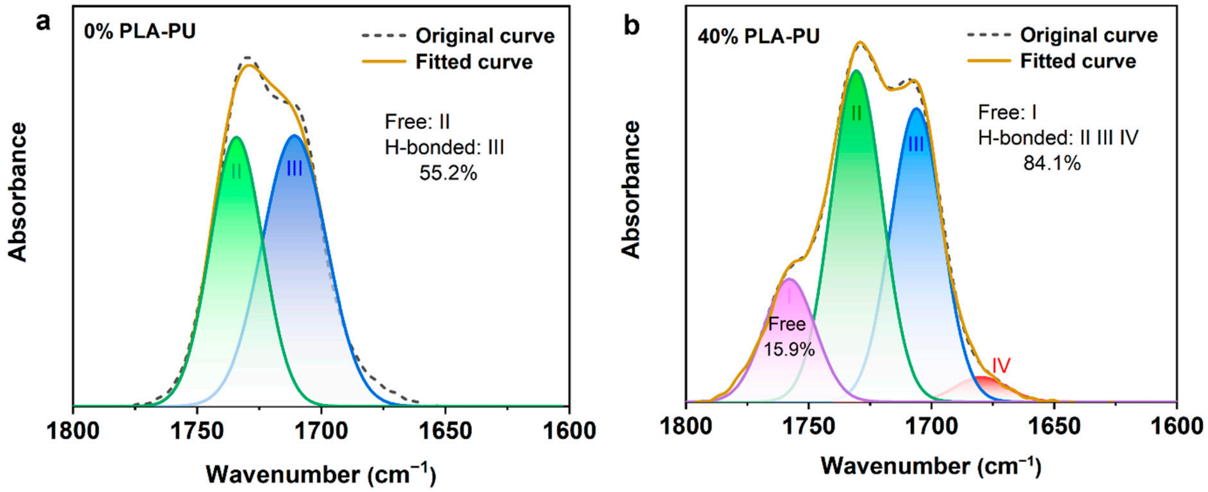

Figure S2. FT-IR spectra of the C=O absorption bands for (a) 0% PLA-PUs and (b) 40% PLA-PUs.

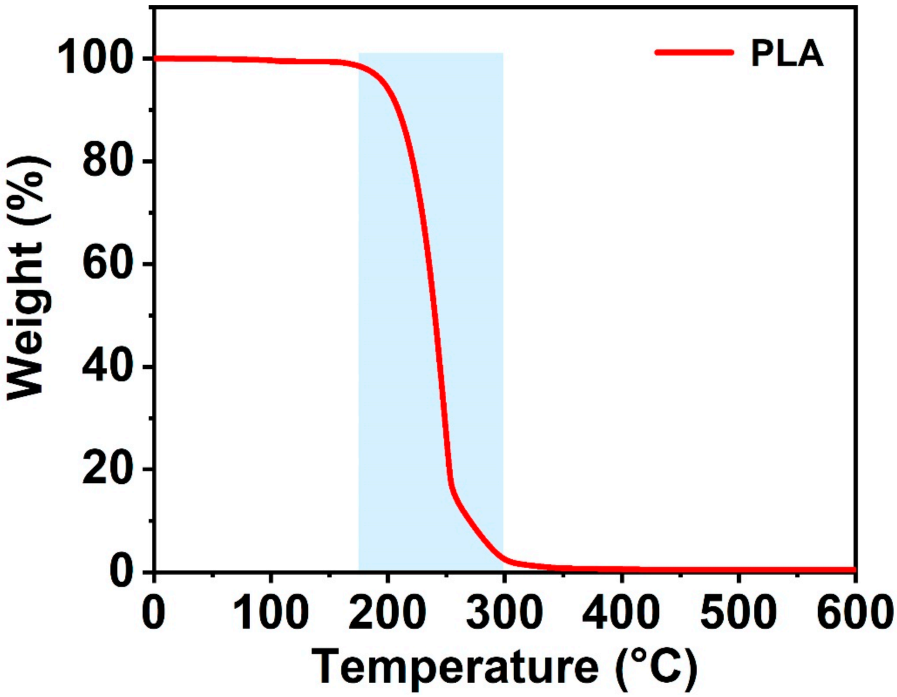

Figure S3. TG curves of PLA-OH (the blue bar area represents the thermal decomposition temperature range).

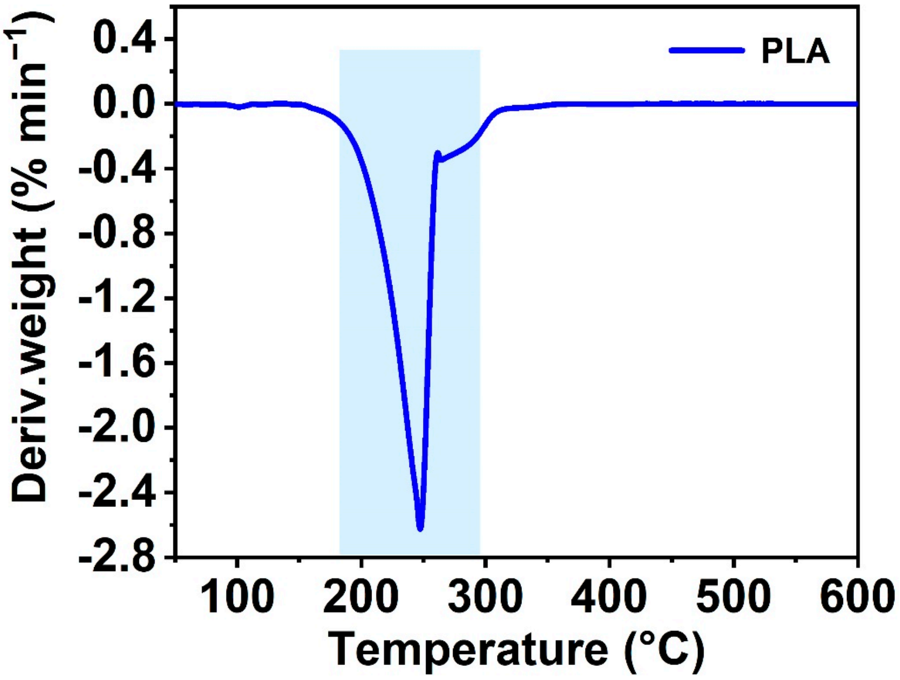

Figure S4. DTG curves of PLA-OH (the blue bar area represents the thermal decomposition temperature range).

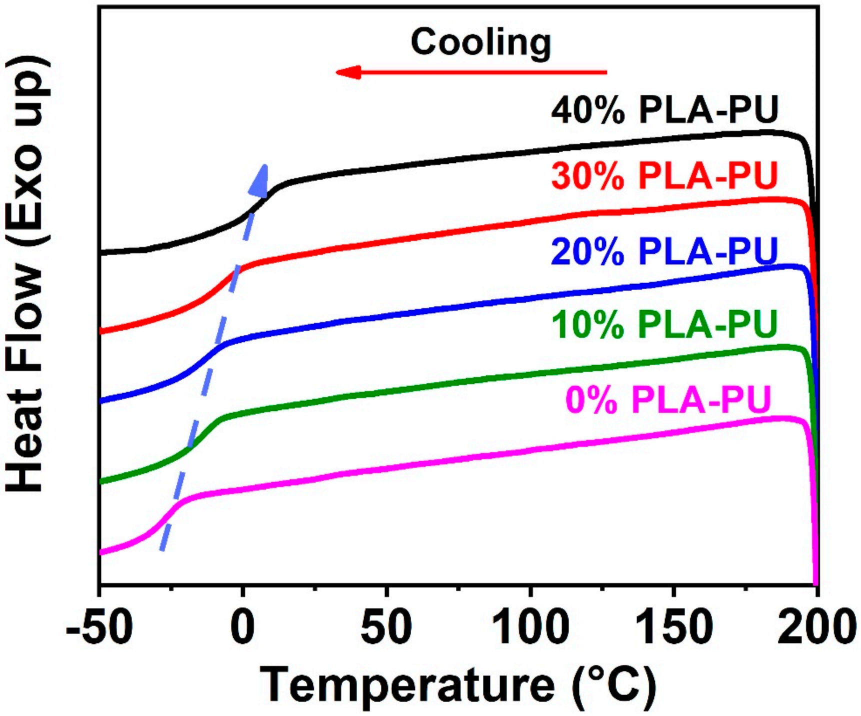

Figure S5. DSC first cooling curves of PLA-PUs.

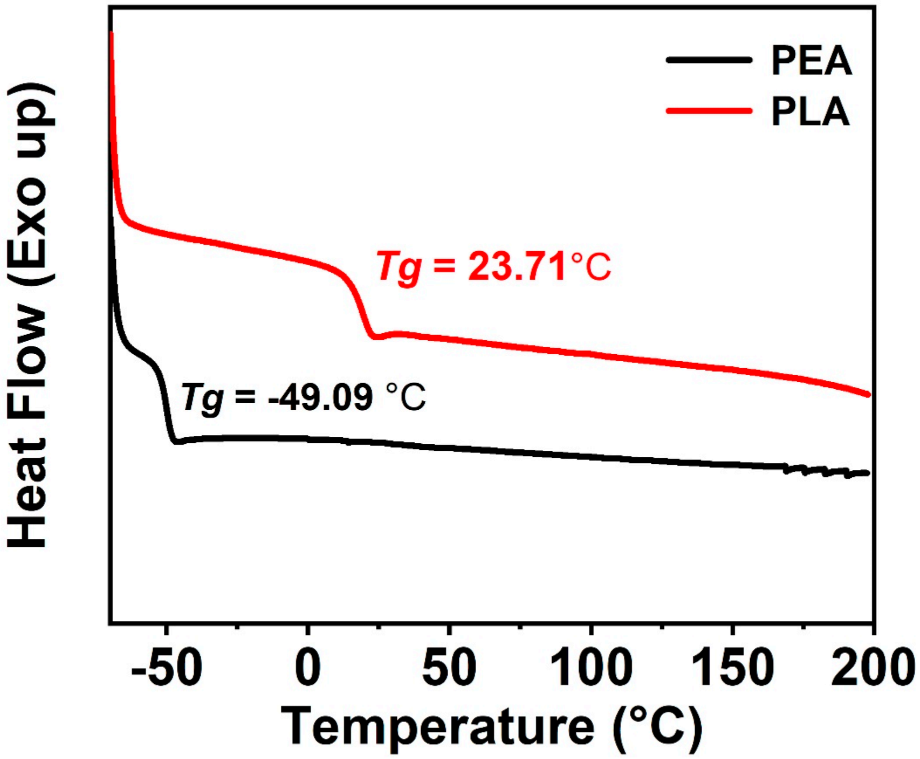

Figure S6. DSC second heating curves of PLA-OH and PEA.

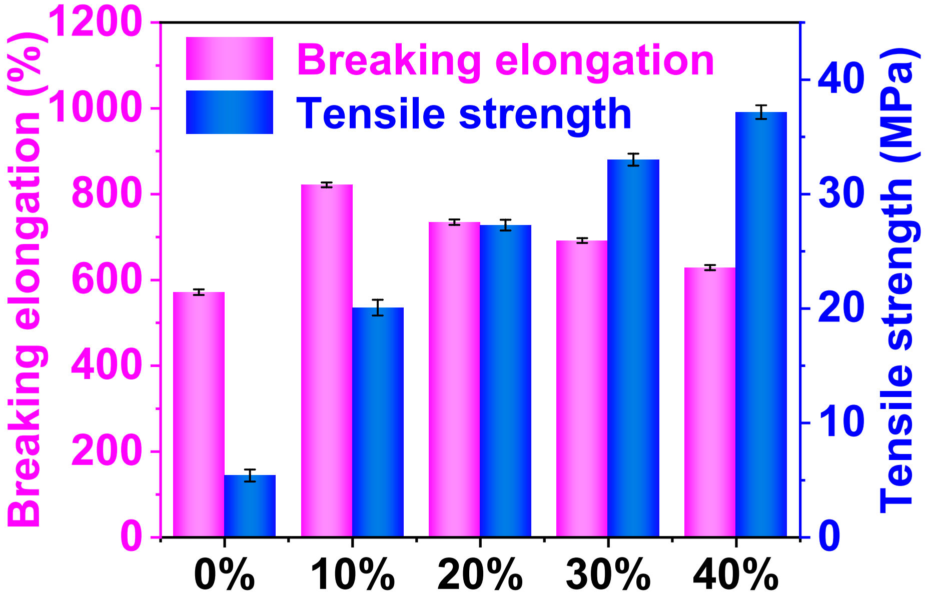

Figure S7. The tensile strength and Breaking elongation of PLA-PUs.

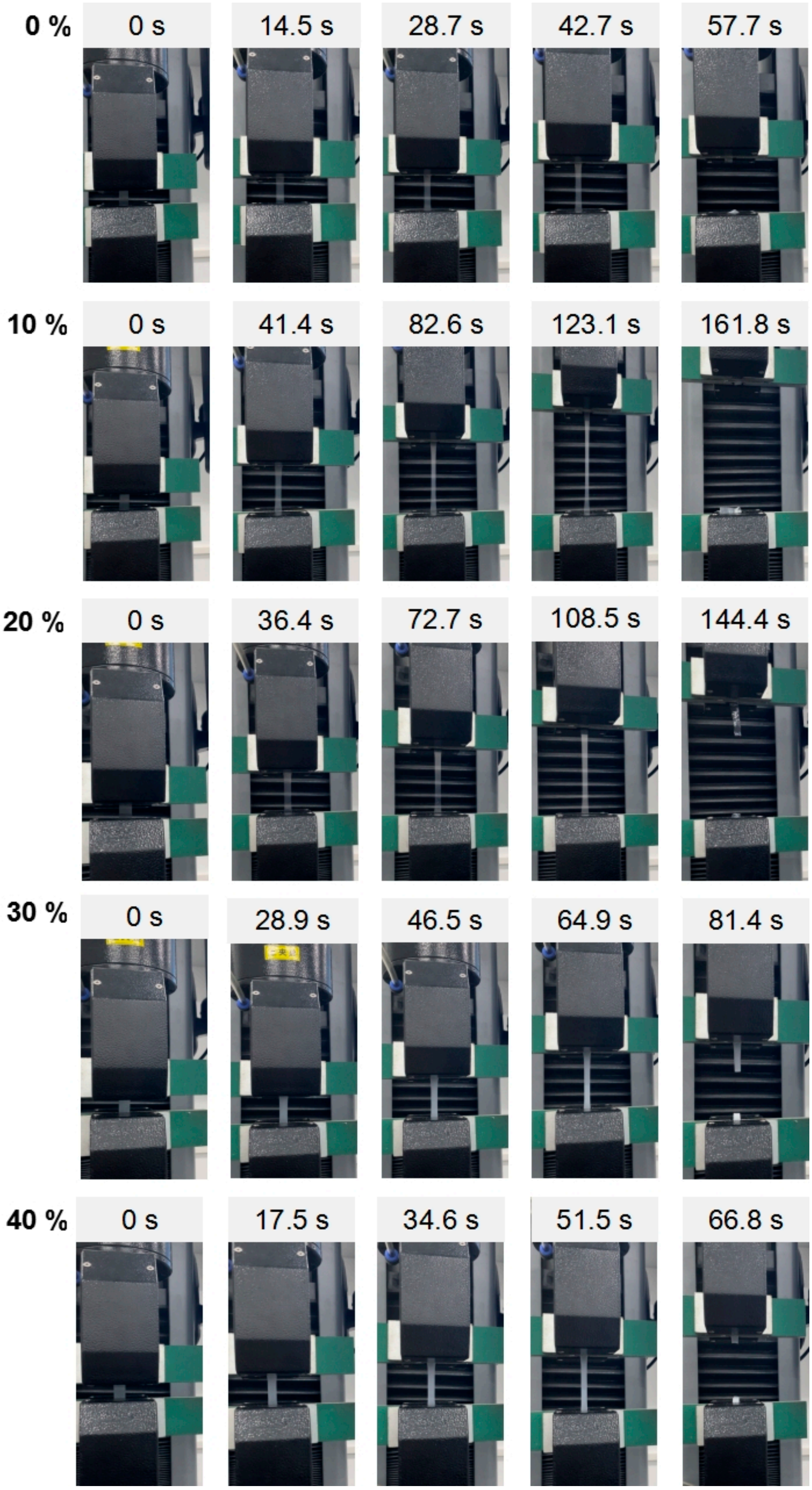

Figure S8. The whole stretching process of 0-40% PLA-PUs with different times.

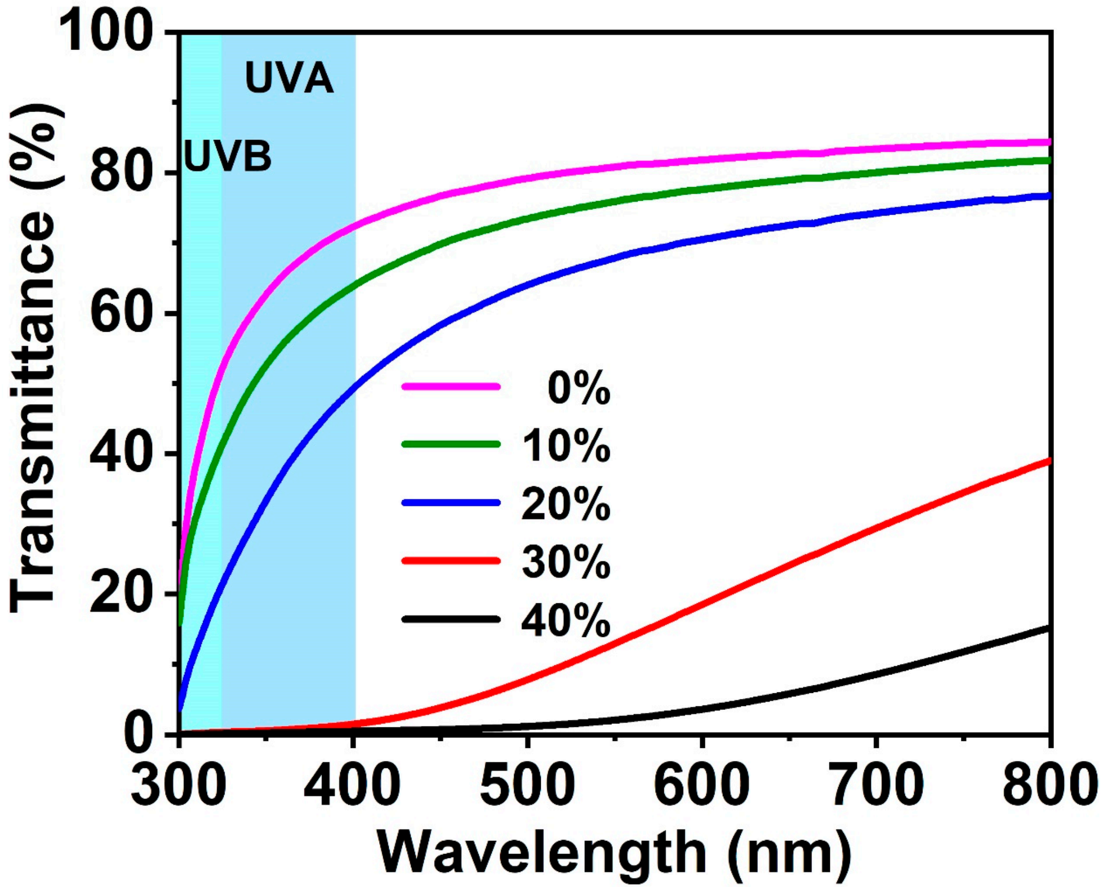

Figure S9. The transmittance of PLA-PUs in the wavelength of 300-800 nm.

Table S1. Formulation of the samples.

Sample	Mn of PLA-OH and PEA polyols	Molar ratio PLA-OH and PEA polyols: MDI: EG
0% PLA-PU	2000	0:1:2:1
10% PLA-PU	2000	0.1:0.9:2:1
20% PLA-PU	2000	0.2:0.8:2:1
30% PLA-PU	2000	0.3:0.7:2:1
40% PLA-PU	2000	0.4:0.6:2:1

Table S2. The molecular weights of PLA-PUs determined by GPC.

Sample	Mn(10^4)	Mw(10^4)	Mw/Mn(PDI)
0% PLA-PU	2.95	5.67	1.92
10% PLA-PU	2.98	5.40	1.81
20% PLA-PU	2.93	5.45	1.86
30% PLA-PU	2.91	5.49	1.87
40% PLA-PU	2.89	5.53	1.91

Table S3. The molecular weight of PLA-PUs after degradation within 6 months determined by GPC

Sample	Degradation time (month)	Mn(10^4)	Mw(10^4)	PDI
0% PLA-PU	initial	2.95	5.67	1.92
	1	2.92	5.59	1.91
	2	2.92	5.57	1.91
	3	2.90	5.49	1.89
	4	2.91	5.46	1.88
	5	2.89	5.48	1.90
	6	2.90	5.37	1.85
10% PLA-PU	initial	2.98	5.40	1.81
	1	2.82	4.95	1.76
	2	2.50	4.77	1.91
	3	1.81	3.81	2.10
	4	1.62	3.73	2.30
	5	1.34	3.41	2.54
	6	1.25	3.12	2.50
20% PLA-PU	initial	2.93	5.45	1.86
	1	2.50	5.01	2.00
	2	2.41	4.86	2.02
	3	1.87	3.95	2.11
	4	1.52	3.42	2.25
	5	1.31	3.11	2.37
	6	1.24	2.86	2.31
30% PLA-PU	initial	2.91	5.49	1.87
	1	2.52	5.11	2.03
	2	2.01	4.83	2.40
	3	1.44	4.01	2.78
	4	1.22	3.58	2.93
	5	1.11	3.11	2.80
	6	1.08	2.83	2.62
40% PLA-PU	initial	2.89	5.53	1.91
	1	2.41	5.22	2.17
	2	2.05	4.76	2.21
	3	1.39	3.94	2.32
	4	1.18	3.38	2.86
	5	1.07	2.91	2.72
	6	1.01	2.55	2.52
